# The lateral line and electrosensory systems of two holocephalans

**DOI:** 10.1038/s41598-025-87499-2

**Published:** 2025-02-28

**Authors:** Laura A. O. Solon, Arnault R. G. Gauthier, Brittany Finucci, Adam T. Downie, Shaun P. Collin, Ian R. Tibbetts, Victoria Camilieri-Asch

**Affiliations:** 1 School of The Environment, The University of Queensland, St Lucia QLD 4072, Brisbane, Australia; 2 Max Planck Queensland Centre (MPQC) for the Materials Science of Extracellular Matrices, Queensland University of Technology, Kelvin Grove QLD 4059, Brisbane, Australia; 3 Centre Sécurité Requin, 97436 St Leu, La Réunion, France; 4 National Institute of Water and Atmospheric Research (NIWA), Hataitai, Wellington 6021, New Zealand; 5 School of Agriculture, Biomedicine and Environment, La Trobe University, Bundoora Vic 3086, Melbourne, Australia; 6 Centre for Biomedical Technologies (CBT), Queensland University of Technology, Kelvin Grove QLD 4059, Brisbane, Australia; 7 ARC Training Centre for Multiscale 3D Imaging, Modelling and Manufacturing (M3D), Queensland University of Technology, Kelvin Grove QLD 4059, Brisbane, Australia; 8 School of Mechanical, Medical and Process Engineering, Queensland University of Technology, Brisbane City QLD 4000, Brisbane, Australia

**Keywords:** Lateral line, Ampullae of Lorenzini, Sensory organs, Chimaeras, Deep sea, Electron microscopy, Ecology, Zoology, Anatomy, Peripheral nervous system, Marine biology

## Abstract

The mechanosensory (lateral line) and electrosensory systems are two important non-visual sensory modalities, especially in low light environments. Despite their importance, these sensory systems have received little attention in deepwater chondrichthyans. Here, we describe the morphological organisation of the peripheral lateral line and electrosensory systems in two species of chimaeras; the pale ghost shark *Hydrolagus bemisi* (Chimaeridae) and the Australasia narrow-nosed spookfish *Harriotta avia* (Rhinochimaeridae), occupying depth ranges of 400–1,100 m and 260–1,278 m, respectively. Using topographic mapping, computed tomography, histology, and scanning electron microscopy, the distribution, abundance, size, and microstructure of lateral line grooves and organs (neuromasts), and ampullary organs (pores, canals, and bulbs) are described. The arrangement of the peripheral sense organs in both these systems may reflect comparable feeding strategies for detecting benthic prey. While the elongated rostrum of *Harriotta avia* is likely used as a sensory probe, providing spatially-resolved information about minute hydrodynamic disturbances and electric fields of potential prey beneath the animal, the arrangement of sense organs in *Hydrolagus bemisi* indicates that this species may rely less on electroreception. The study compares the morphology and provides information on the relative importance of two (non-visual) sensory modalities in two demersal holocephalans that remain vulnerable to anthropogenic disturbances.

## Introduction

Cartilaginous fishes (Chondrichthyes) are the oldest living group of jawed vertebrates. This fish group includes two main radiations – chimaeras (Holocephali), and sharks, skates, and rays (Elasmobranchii) – that diverged about 420 million years ago, between the Middle Ordovician and the Early Devonian^1–5^. Most holocephalans inhabit depths beyond 200 m, making them logistically more difficult to observe *in situ* and to access for sampling^6–8^. Species from this sub-group remain the least studied among chondrichthyans globally, and there is still limited information about their biology, behaviour and life histories^7,9^. Yet, such knowledge is important to better predict potential anthropogenic impacts to their populations in future, as resource exploration and exploitation increasingly expand in deeper parts of the world’s oceans^6^.

Deepwater chondrichthyans live under extreme environmental conditions, such as high hydrostatic pressures, low temperatures, reduced dissolved oxygen levels, and have evolved certain morphological traits that enable them to thrive in the deep-sea (> 200 m)^6,10^. Many deepwater chondrichthyans have an enlarged medulla oblongata (the brain region receiving mechano- and electrosensory inputs) relative to the total brain size, which suggests that they rely more on mechano- and electroreception than other senses^11^. In addition to an enlarged medulla, holocephalans have a relatively larger cerebellum (the brain region receiving various information, including sensory input related to body movement within a given spatial georeference), which potentially reflects a specific adaptation to a deepwater demersal habitat, where species that swim slowly above the benthos move through largely two-dimensional environments^12^. Although previous studies have reported adaptations in the visual and chemoreceptive systems in holocephalans^13^ (and reviewed by^6,14^), there is limited information on other sensory modalities. Specifically, few studies have described the morphological organisation of the lateral line and electroreceptive systems in holocephalans, to establish how they may be used, and thus better understand their sensory ecology.

In fishes, underwater vibrations (or oscillatory motions: e.g., sound waves) and water movements are mediated by the mechanosensory (or *acousticolateralis*) system. While sounds and other (high or low) frequency underwater vibrations are detected by the inner ear^14^, hydrodynamic disturbances and low frequency vibrations are primarily detected by the lateral line^15^. Fish use the (mechanosensory) lateral line for localising food^16,17^, conspecifics and objects^18^ within their immediate environment, for orientation^19–21^, for social communication^22^ and for predator avoidance^23,24^. A network of canals encloses the sensory organs, called neuromasts, that line the medial canal walls. Neuromasts comprise groups of ciliated sensory (hair) cells surrounded by non-sensory (support and mantle) cells^25^. The sensory hair cells are distinguishable as they bear a well-defined ciliary bundle on their apical surface, which consist of a long kinocilium and a group of stereocilia, covered by a gelatinous cupula^25,26^; while non-sensory cells bear apical microvilli^23^. In chondrichthyans, the lateral line system detects short-range stimuli, from a few centimetres above the skin surface for non-pored canals^24,27^ to a few body lengths for pored canals^28,29^, with canal neuromasts typically detecting low-frequency (< 200 Hz) water displacements^23,28,30^. However, most deepwater holocephalans possess continuous open canals, called grooves, over their head and along their lateral flanks^31^, except shallower species such as callorhinchids (plough-nose chimaeras) that possess tubular canals like elasmobranchs^14^. These lateral line grooves (LLGs) are homologous to lateral line canals in elasmobranchs. Holocephalan species studied to date show similar patterns in the distribution of their LLGs^14,32,33^, which are typically supported by wide C-shaped cartilaginous rings^28,30^. Only Von Lubitz (1981)^34^ and Fields (1982, 1993)^35,36^ have described the detailed ultrastructure of the sensory organs (neuromasts) in the rabbit fish *Chimaera monstrosa* and the spotted ratfish *Hydrolagus colliei*, respectively.

Another non-visual system, passive electroreception, is the ability to detect weak bioelectrical fields, generated by muscle and neural activities. This modality serves numerous biological functions in chondrichthyans; including locating prey^37–45^, detecting predators and conspecifics^46–49^, and in communication^46,50^ and navigation^37,52–52^. The electrosensory (or ampullary) organs are called ampullae of Lorenzini. Each ampulla comprises three components; an ampullary pore, canal, and bulb, which are filled with an electro-conductive gel. Ampullary pores, visible externally on the skin surface, are mainly situated on the head, from the tip of the rostrum or snout (the anterior prolongation of an animal’s head) to the first gill slit in sharks^53–55^ but extend on to the pectoral fins in batoids^56–60^. Ampullary canals connect the ampullary pores to their corresponding bulbs. These bulbs are small ‘sacs’ grouped into gelatinous clusters (3–5 in sharks, 4–6 in batoids), and are located in specific areas, subdermal or deeper within the head^53–60^. Each ampullary bulb usually connects to the skin surface via one canal, which length depends on the distance between the cluster they extend from and the skin area where they terminate as pores. Internally, within the bulb, each canal leads to a large chamber (the ampulla proper), often divided into several sensory chambers that are lined with sensory epithelium and sometimes sub-divided into even smaller compartments (alveoli or sub-chambers) by small non-sensory epithelial protrusions or folds called septa^25,61^.

Electrosensitivity is defined as the ability to detect weak bioelectric fields, where the abundance and distribution of ampullary pores can improve the ability to spatially resolve electrical stimuli during foraging^42,62^. The electrosensory system operates at short range (< 1 m), where individual electroreceptor cells detect potential differences between their apical and basal membranes (beyond a certain threshold of < 5 nV.cm^− 1^), which triggers a signal that is conveyed to the brain via an afferent nerve (the anterior lateral line nerve)^43^. The sensory chambers of holocephalan ampullary bulb are dactyliform (finger-shaped), with elongated alveoli (e.g. in *Hydrolagus colliei*^63^) and can detect stimuli of < 0.2 µV.cm^− 1^ ^63^ compared to the nanovolt-sensitivities reported for elasmobranchs, such as the scalloped hammerhead shark *Sphyrna lewini* and the sandbar shark *Carcharhinus plumbeus*^43^. To date, there remains little information on the holocephalan ampullary system, where electroreception has been shown to play a role in feeding and geomagnetic orientation^63,64^. Despite potentially unique morphological and physiological characteristics, only one recent study has described the electrosensory system in this group, e.g. in the rabbitfish *Chimaera monstrosa*^65^.

Investigations of both lateral line and electrosensory systems in holocephalans are limited to *Chimaera monstrosa*^34,65^ and *Hydrolagus colliei*^63^, occupying depth ranges of 50–1,742 m and 0–1,029 m, respectively^66^. Here, we extend this approach to two additional species with a combined depth range of 260–1,278 m^66,67^, i.e., the pale ghost shark *Hydrolagus bemisi* (Chimaeridae) and the Australasia narrow-nosed spookfish *Harriotta avia* (Rhinochimaeridae) (recently described as a new species that is distinct from the narrownose chimaera *Harriotta raleighana*^67^ with a depth range of 350–2,600 m^66^). These study species were targeted to examine sensory adaptations of this lesser-known and unique group of cartilaginous fishes to deeper environments. Indeed, most of the biomass for *Harriotta* spp. is found deeper than *Chimaera* spp. and *Hydrolagus* spp.^68^.

Temperature, light, hydrostatic pressure, currents, water density and sound are all biophysical factors that change with increasing depth and have critical impacts on the peripheral sense organs of deep-sea fishes [see^10^ for review]. Some deep-sea teleosts (or bony fishes) possess widened and membranous lateral line canals that provide as much as a 100-fold increase in sensitivity compared to narrow canals^69^, or increase the height of their cupula and/or raise the superficial neuromasts onto a small papilla (and therefore above the boundary layer) to enhance sensitivity to slow flows and low frequency stimuli^70^. The low abundance of food and the changes in the biophysical characteristics of electric fields at extreme depths (i.e., the peak-to-peak value of the natural electric field decreases), may be a selective pressure that also led to specific adaptations in the peripheral electrosensory system of chondrichthyans^71^. As has been established in shallow water fishes^45,72,73,74^, we hypothesised that both study species would show phylogenetic similarities in the gross morphological organisation of these two peripheral sensory systems, yet would display interspecific variations reflecting potential ecological adaptations linked to their feeding strategy and depth habitat. This study aims to: (1) increase our knowledge of non-visual sensory systems in these two deepwater species, comparing their morphological organisation in order to predict aspects of their sensory ecology, and (2) uncover adaptations that specifically enable them to overcome the biophysical constraints of their environment by establishing correlative evidence to support structure-function relationships.

## Methods

### Ethical sourcing of specimens

Specimens were imported to the Queensland University of Technology (QUT) under Biosecurity Import Conditions (BICON) permit for ‘Standard goods’ 005253646. Tissue samples used for this study (skin and ampullary clusters) were accessed via Dr. Camilieri-Asch (VCA) at QUT, where specimens were originally received and stored. The research was conducted both at QUT and The University of Queensland (UQ) under QUT ethics approval for tissue use (Approval no. 2000000191) and the UQ Ethics Permit (Approval no. 022/AE000100). This research conformed to Australian law. It was carried out in strict accordance with the ethical guidelines of both institutions (QUT and UQ), following the Australian Code of Practice for the Care and Use of Animals for Scientific Purposes (National Health and Medical Research Council, 2013). The authors complied with the ARRIVE guidelines 2.0.

Specimens were collected on Chatham Rise, New Zealand, during a fishery-independent survey in January 2021 at depths from 631 to 663 m. Three mature specimens (2 female/1 male) from two species of chimaeras were used in this study; the pale ghost shark *Hydrolagus bemisi* (*n* = 3; chimaera length CL, snout to posterior end of supracaudal fin^75,76^ between 66.4 and 78.9 cm) and the narrownose chimaera *Harriotta avia* (*n* = 3; CL = 67.1–92.3 cm). The heads of specimens were bisected and were either retained frozen or fixed in 10% neutral buffered formalin (NBF) (Table 1). The frozen heads were solely used for mapping and pore counting. For fixed specimens, the brain was exposed by performing a small cranial excision (1–2 cm diameter) at the top of the neurocranium (above the telencephalic region) to facilitate and ensure prompt and optimal internal (brain) fixation for a separate study. All specimens were kept in either their fixative solution or frozen, and then stored in a fridge (4˚C) or freezer (-20˚C), respectively, prior and following shipment to Australia.

**Table 1:** List of specimens donated for this study and their morphometrics. CL, chimaera length (snout to posterior end of supracaudal fin) in millimetres; BW, body weight in grams; NBF, neutral buffered formalin.

Scientific name	Common name	Specimen	Sex	CL (mm)	BW (g)	Maturity	Catch depth (m)	Fixed or frozen
*Hydrolagus bemisi*	Pale ghost shark	GSP1	M	664	1564	mature	648–663	10% NBF
		GSP2	F	789	2886	mature	631–637	10% NBF
		GSP3	F	743	2218	mature	631–637	Frozen
*Harriotta avia*	Australasia narrow-nosed spookfish	LCH1	M	671	796	mature	648–663	10% NBF
		LCH2	F	923	2524	mature	648–663	10% NBF
		LCH3	F	864	2148	mature	648–663	Frozen

### Morphological assessments

#### Lateral line groove, ampullary pore and canal distribution

Heads were transferred and rinsed in 1 M phosphate buffered saline (PBS) solution for 30–45 min prior to dissection. The skin was divided into two main pieces (left/right or dorsal/ventral) depending on whether the species’ head morphology was laterally (*H. bemisi*) or dorso-ventrally (*H. avia*) flattened. The skin of each specimen was removed by carefully peeling and cutting away the connective tissues beneath the dermis. Each skin sample was placed onto a lightbox (ultra slim LED light pad, X-Press It^®^) to map both the lateral line open canals or lateral line grooves (LLGs), and the ampullary pore openings and their corresponding canals, with light projected from beneath. The outlines of the skin samples and the position of LLGs and ampullary pores were drawn onto A4 transparent films with permanent markers of different colours. The relative diameter and course of grooves and ampullary canals for both lateral line and ampullary systems, respectively, were also recorded when clearly visible, to represent them in diagrams for qualitatively descriptions. Transparent films were blotted dry with paper towel, and stored until further processing. Once the mapping was completed, all films were scanned and digitalised, to graphically edit the resulting distribution maps using ProCreate (version 5.3).

#### Ampullary pore abundance

To quantify the number of ampullary pores, the drawings corresponding to each specimen’s head were divided into dorsal/ventral and anterior/posterior areas, as follows; (1) a horizontal line from the tip of the rostrum or snout through the middle of the eyes to the first gill opening distinguished the dorsal and ventral areas, and (2) a vertical line through the middle of the eyes distinguished the anterior and posterior areas. This approach was used to report ecologically-relevant differences in pore counts between study species.

#### Ampullary pore size

The skin samples from fixed specimens (*n* = 2 per species) were immersed in 1 M PBS solution for a minimum of 45 min, prior to being placed onto the light box previously described, where the location of ampullary openings were imaged using a stereo-microscope (Nikon DS-Fi2) mounted on a boom stand. Images were captured systematically, following a grid-cell sampling method, with the software NIS-elements (D4.51.00 64-bit) using the following parameters; x4 calibration; 8-bit fast focus; 8-bit image quality. For each image captured, only the diameter of ampullary pores that were in focus were measured using ImageJ software (version IJ1. 46r, National Institute of Health (NIH), USA). An average of 130 pores (*Hydrolagus bemisi*) and 190 pores (*Harriotta avia*) were measured per specimen of each species. As specimens had markedly fewer and bigger pores on the dorsal surface, proportionally, fewer pores were measured on the dorsal than the ventral side, representing the natural distribution. Average ampullary pore diameter for each head region, specimen and species was reported as mean values (± standard deviation, SD).

### Micro-computed tomography (µCT) imaging

Existing µCT data (from specimens stained with a 6% iodine-based (I_2_KI) in 10% NBF solution, following Camilieri-Asch et al.. (2020)^77^) were available for both species to visualise the extent and position of both LLGs and ampullary clusters *in situ.* The 3D rendered data (voxel size between 25 and 60 μm) enabled the collection of descriptive information that were useful to adjust the position of LLGs and ampullary pore openings, as well as the shape and size of ampullary clusters, relative to the head morphology. Specifically, 2D (slice) images of the lateral line grooves or canals and ampullary clusters facilitated the generation of a more accurate digital representation of the position of external and associated internal structures, for the LLG and ampullary systems, respectively. All visualisations and images were captured using Avizo 2021.2.

## Histology

For the LLG system, skin tissue samples bearing externally visible LLGs (*N* = 12 per specimen, using *n* = 2 fixed specimens per species, so 24 samples per species) were collected across the head under a dissecting microscope (Nikon DS-FI2 + SMZ745T), and decalcified in 10% EDTA (ethylenediaminetetraacetic acid) at 37 °C (pH 7.4) for a minimum of three days using a multifunctional microwave tissue processor (KOS Microwave HistoStation). Decalcification levels were checked daily by scout scanning of each sample using X-ray micro-computed tomography (Scanco Medical µCT 50 cabinet scanner).

For the ampullary system, single ampullae of Lorenzini (*N* = 20 per specimen; 40 per species) that lay deeper in ampullary clusters within the rostrum were also dissected, rinsed in distilled water for 30 min and stored in 70% ethanol until further processing (no decalcification was required).

For both systems, decalcified (LLG) and dissected (ampullary) samples were then prepared for paraffin embedding using a tissue processor (Leica ASP300S) following an overnight protocol that included a series of dehydration steps (70%, 90%, 3 × 100%, EtOH and 3x xylene at 37˚C) then gradual infiltration steps in paraffin wax at 60˚C. Infiltrated samples were manually embedded in paraffin wax on an embedding station (Leica Histocore Arcardia H); half were embedded in a transversal orientation and half in a longitudinal orientation. Freshly embedded blocks were left to set on the cooling plate at -4˚C (Leica Histocore Arcardia H) for 20 min, then stored in sealed plastic containers.

Paraffin-embedded samples (blocks) were sectioned using a semi-automatic microtome (Leica RM2135). For each block, semi-thin paraffin Sect. (5 μm) were collected and mounted on poly-lysine coated microscope slides (Thermo Fischer Scientific), then cured for 2 h in the oven at 60˚C. All slides were stained with solutions of Harris haematoxylin (Harris haematoxylin mercury free, POCD Scientific, VWRC351945S) and 0.25% ethanol-based eosin (Eosin-Y Sigma-Aldrich, 230251; or Eosin-Y, Glenthan Life science, GT2503), following an optimised protocol (see Supplementary Table S1) programmed into an autostainer platform (Leica ST5010 autostainer XL).

The haematoxylin and eosin (H and E) stained slides were scanned with a slide scanner (3DHISTECH, Panoramic Scan II) and digitised images were used to: (1) qualitatively assess the morphological and structural organisation of the peripheral sense organs (i.e., LLGs and neuromasts, and ampullary pore openings, canals and sensory structures), and (2) quantify anatomical and cellular metrics (LLG width and ampulla diameter). Images of LLGs, ampullary canals and ampullae of Lorenzini, as well as their respective sensory epithelia were captured using CaseViewer software (CaseViewer 2.4 for Windows). All quantitative data were collected using Image J, and reported as mean values (± SD).

## Scanning electron microscopy

To visualise the sensory epithelial surface of both peripheral sense organs and describe their organisation and ultrastructure, respective tissues were prepared for scanning electron microscopy (SEM) as follows. For the LLG system, skin tissue samples bearing visible grooves (*N* = 40 per species) were dissected from the head of each fixed specimen (*n* = 2) and kept in 10% NBF until further processing. For the ampullary system, individual ampullae (ampullary bulbs and a portion of their corresponding canal) were collected (*N* = 20 per species) from the rostral cluster of each fixed specimen (*n* = 2) and kept in 10% NBF until further tissue processing.

Prior to SEM sample preparation, a third of the ampullae were kept whole (to image the external bulb surface), while the remaining ampullae were sonicated for 15 min and cut in half (either longitudinally or transversally), to remove the gel that fills the ampullary canal and the ampullary bulb. This approach enhanced the visualisation of different morphological areas within the ampullary bulb (e.g., ampulla proper, sensory chambers).

SEM sample preparation consisted of the following steps: washes in 0.1 M phosphate buffer, fixation in 2.5% glutaraldehyde in 0.1 M phosphate buffer (for 1 h for the ampullae), post-fixation in osmium tetroxide (OsO_4_) for 1 h (ampullae) and up to 2 h (skin) to avoid a loss of samples as they were originally transparent, further buffer washes, dehydration through an ascending ethanol series (20%, 30%, 40%, 50%, 60%, 70%, 80%, 90% x2 and 100% x2 EtOH, ) for 10 min each (ampullary samples), or 30 min each (skin samples with visible LLGs), and evaporative drying in 100% HMDS (hexamethyldisilazane) overnight. Each processed sample was mounted onto a 12-mm diameter aluminium stub using double-sided carbon tape, with whole ampullae having been bisected either longitudinally or transversally before mounting. All mounted samples were coated with platinum to 4 nm thickness (2 × 2 nm at different angles to cover samples well, given their complex spatial geometry).

Images were captured with a field emission SEM (Tescan MIRA3), operating at 5kv/8 mA electron beam and secondary electron (SE) detector at a 6–8 mm working distance. The resulting electron micrographs were used to qualitatively describe the spatial (surface) ultrastructure of the LLGs and neuromasts, and ampullary structures (pores, canals, ampullae and sensory chambers). These qualitative 3D surface data were used to complement 2D histological descriptions and confirm the morphology and average number of sensory chambers present in the ampullae of Lorenzini for each species.

### Statistical analyses

Statistical analyses were performed in RStudio 2023.12.1 + 402 (R Core Team 2020) to test for inter- and intraspecific differences in several morphological parameters; lateral line groove diameter, ampullary pore count, and ampullary pore, canal and bulb diameters. Quantitative data were reported as mean values (± SD). For each parameter, mean values were statistically compared using unpaired (two-samples) Wilcoxon tests (Wilcoxon Rank Sum Test) if the data were non-parametric, or an unpaired (two-samples) t-test if they were parametric, with 95% confidence intervals (α = 0.05).

## Results

### Gross morphological organisation of the lateral line system

**Fig. 1:**
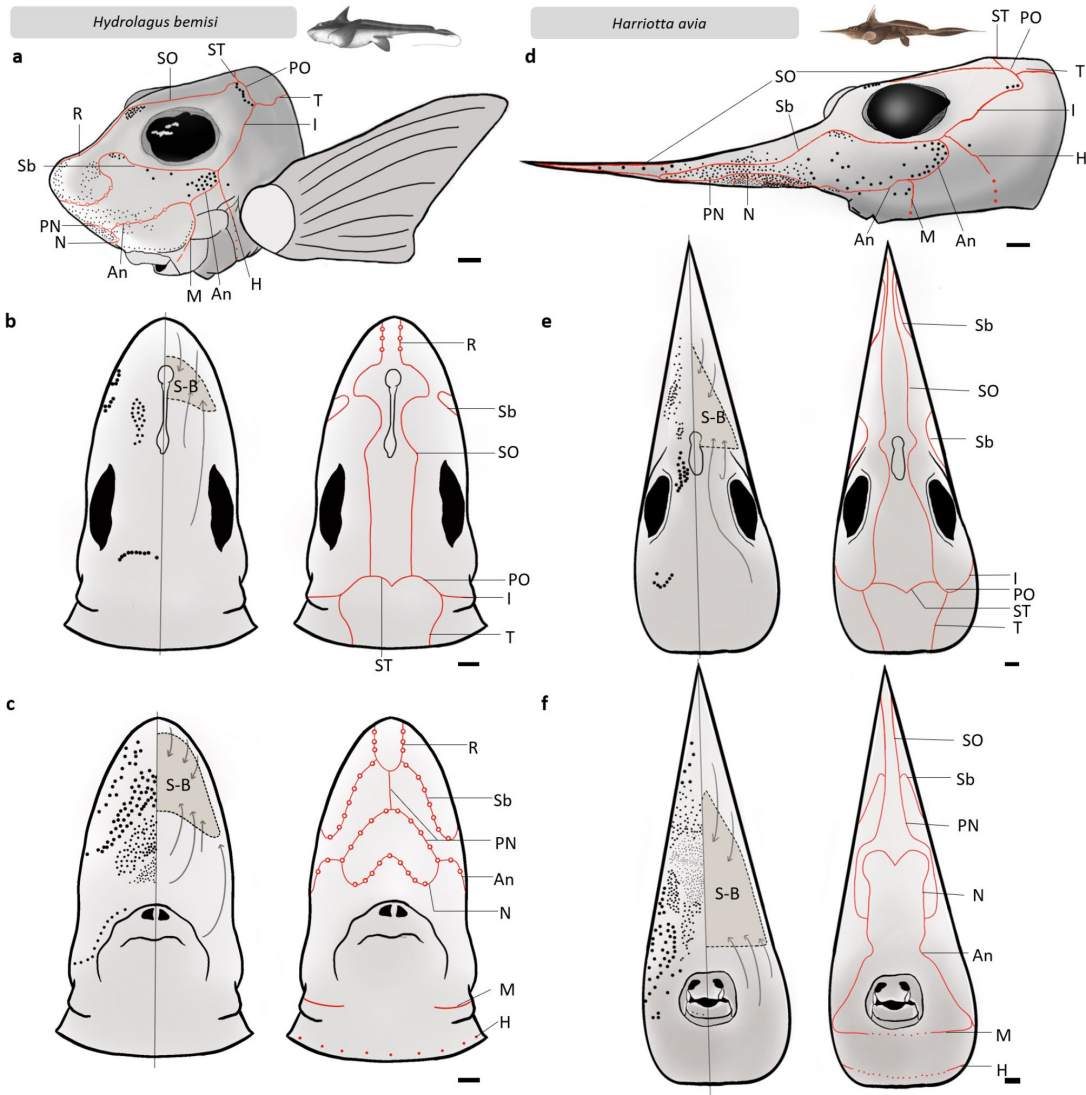
Schematic drawings representing the morphological distribution of peripheral sensory features of the lateral line and electrosensory (ampullary) systems present on the head of *Hydrolagus bemisi* (left, **a**-**c**) and *Harriotta avia* (right, **d**-**f**); in lateral (**a**,** d**), dorsal (**b**,** e**), and ventral (**c**,** f**) views. In *H. bemisi*, the lateral line system is characterised by two types of open canals or grooves: lineate (or linear) grooves represented by plain red lines, and sacculate (or circular) grooves, which are linear grooves that bear large dilations represented by red circles. Possible neuromasts are represented by red dots. Only lineate grooves were observed in *H. avia*. The electrosensory system is represented in black, with individual ampullary pores depicted as dots. Ampullary clusters are represented as grey zones, labelled (S-B) and delineated by a dashed grey line, in dorsal (**b**, **e**) and ventral views (**c**,** f**). The main orientations (or directions) of associated ampullary canals are represented by grey arrows projecting from the main pore areas to the cluster where most ampullary bulbs are located. Please note that pore size and cluster morphology are only indicative of the three-dimensional observations made and were arbitrarily represented to scale. An, angular groove; H, hyomandibular groove; I, infraorbital groove; M, mandibular groove; N, nasal groove; PN, prenasal groove; PO, postorbital groove; R, rostral groove; Sb, suborbital groove; SO, supraorbital groove; ST, supratemporal groove; T, trunk groove; S-B, superficial ophthalmic buccal cluster. Scale = 1 cm.

Both species show a very similar, bilaterally symmetrical distribution of opened lateral line canals called lateral line grooves (LLGs) across the head (Fig. 1), with one exception. Interestingly, there is a slight difference between the left and the right sides for two out of the three specimens in both species, where the hyomandibular groove arises from the infraorbital groove below the eyes on the right side of the head. The pale ghost shark *Hydrolagus bemisi* and narrownose chimaera *Harriotta avia* possess comparable ‘primary canal’ types, which are thin, long grooves. However, there are noticeable interspecific differences in the distribution, organisation and appearance of these grooves (Fig. 1). Thereafter, the nomenclature used to describe the LLGs is based on the most recent nomenclature applied to chimaeras wherever possible. We provide the nomenclature currently used for other chondrichthyans in brackets, when different, to link findings from this study to previous literature (please also see Supplementary Table S2).

*Hydrolagus bemisi* possess a supratemporal (ST) (or aural) groove running transversally on the posterodorsal part of the head, leading to paired postorbital (PO) (or occipital) grooves on either side of the head, at the upper posterior corner of the eye (Fig. 1a, b). The postorbital grooves then split into the infraorbital (I) (or orbital) grooves that continue ventrally behind the eye and project anteriorly towards the mouth, and the trunk (T) (or posterior, or body) grooves that run along the dorsal side of the flank (Fig. 1a, b). Paired supraorbital (SO) (or cranial) grooves run between the eyes, from the intersection of the supratemporal (ST) and the postorbital (PO) grooves, towards the anterior part of the head and the rostrum, where they become the rostral (R) grooves (Fig. 1a-c). The rostral (R) grooves join the prenasal (PN) (or subrostral) groove(s) on the ventral rostrum (Fig. 1a, c) (merging into a single groove that divides again anteriorly in *H. bemisi*; Fig. 1c). The suborbital (Sb) (or infraorbital) grooves that run below the eyes, join either the rostral (R) grooves in *H. bemisi*, or the SO-PN grooves in *H. avia* (Fig. 1d, f). On the ventral side of the rostrum, the prenasal (PN) grooves lead to the nasal (N) groove located anteriorly to the nostrils and join the (paired) intersections between angular (An) grooves that run laterally between the mouth and the suborbital (SO) grooves, and the prenasal (PN) grooves coming from the snout (Fig. 1c). The angular (An) grooves split into the mandibular (M) (or oral) groove behind the top corner of the mouth, and split again to form the hyomandibular (H) (or jugular) groove below the eye, at the junction with the infraorbital (I) and the suborbital (Sb) grooves. The hyomandibular (H) groove runs towards the ventral side in front of the pectoral fins (Fig. 1a, c).

Several differences are noted in the distribution of grooves in *H. avia* (Fig. 1d-f) when compared to *H. bemisi*. These include: (1) the absence of a rostral (R) groove joining (or between) the suborbital (SO) and prenasal (PN) grooves; (2) non-joined prenasal (PN) grooves joining the suborbital (SO) grooves; (3) suborbital (Sb) grooves sitting relatively closer to the eyes, on the dorsal side, likely due to a different head morphology (longer snout) in *H. avia*; and (4) a nasal (N) groove also originating at the junctions of the prenasal (PN) and angular (An) grooves, but located more rostrally (further from the nostrils), at mid-rostrum length (Fig. 1f). Another morphological difference is the presence of sacculate grooves^35,63,36^ that bear large, regularly spaced, round dilations, at the tip of the snout and the entire ventral rostrum in *H. bemisi* (Fig. 1a- c), as opposed to lineate, i.e., linear, narrower grooves^35,63,36^ observed on the rest of the head and body. These sacculate grooves are not present in *H. avia*, which possesses solely lineate grooves. The sacculate grooves observed in *H. bemisi* in this study are similar to a closely-related species (*Chimaera monstrosa*).

### Micro-structure of lateral line grooves and neuromasts

**Fig. 2:**
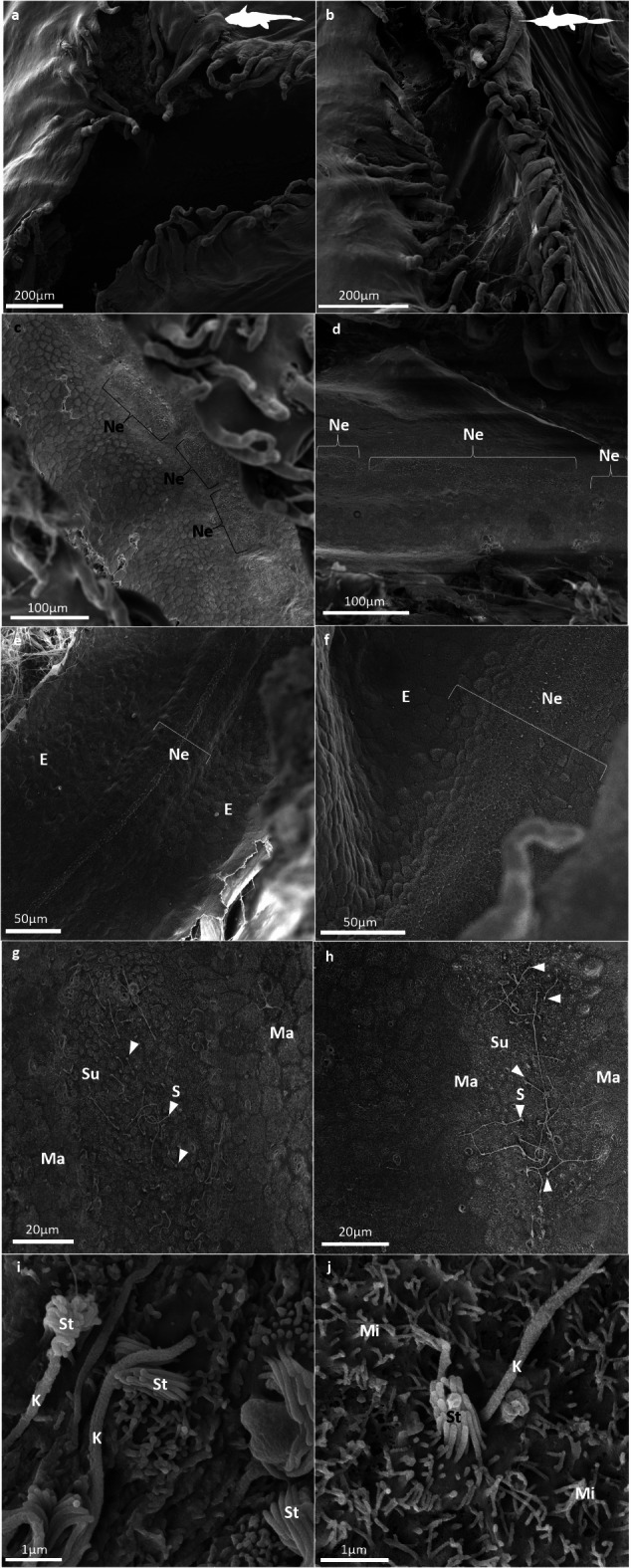
Scanning electron micrographs showing (**a**, **b**) the opening of the lateral line grooves (LLGs), (**c**, **d**) the non-continuous neuromasts (Ne, or lateral line organs) visible on the medial part of the basal wall of grooves, (**e**, **f**) the sensory epithelium constituting the lateral line sensory organ or neuromasts (Ne), which mantle cells (Ma) delineate and separate from the non-sensory epithelium (E), (**g**, **h**) the ultrastructure of the neuromasts (Ne), dotted with sensory cells (S; white arrows) that are surrounded by support cells (Su), all of which are delineated by mantle cells (Ma), (**i**, **j**) the ultrastructure of sensory cells, which apical surface bears a typical, well-defined ciliary bundle, consisting of a single, elongated kinocilium (K) next to a group of stereocilia (St). Sensory cells are surrounded by support cells, which apical surface bears numerous short microvilli (Mi), in both study species – *Hydrolagus bemisi* (left: **a**, **c**, **e**, **g**, **i**) and *Harriotta avia* (right: **b**, **d**, **f**, **h**, **j**).

**Fig. 3:**
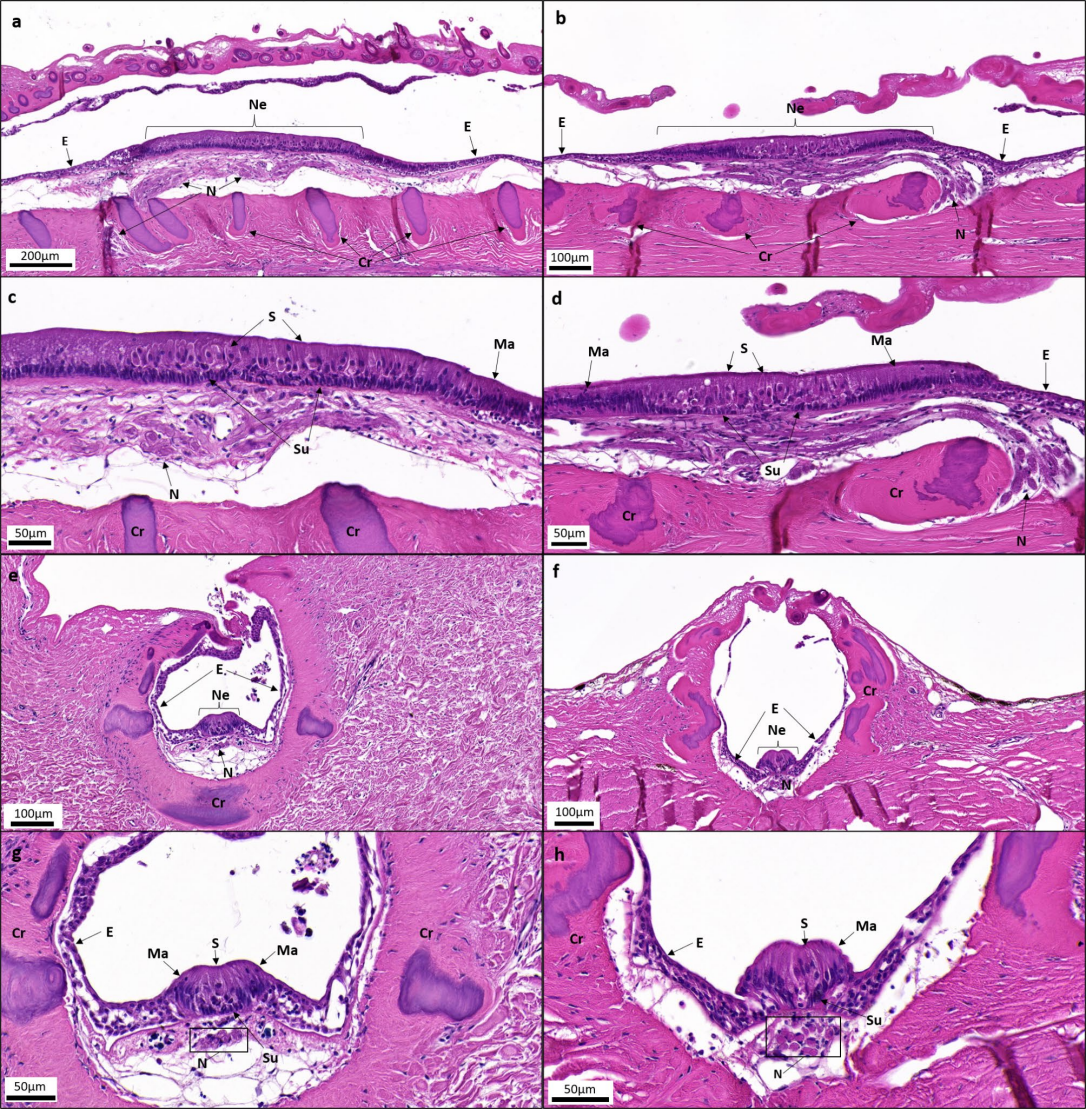
Light micrographs showing a selection of histological sections of the lateral line grooves and neuromasts in *Hydrolagus bemisi* (left: **a**, **c**, **e**, **g**) and *Harriotta avia* (right: **b**, **d**, **f**, **h**), stained with a modified Hematoxylin and Eosin-Y solution (see methods section for staining protocol used). Each micrograph shows (**a**, **b**) the lateral line grooves in longitudinal view, (**c**, **d**) the neuromasts in longitudinal view, (**e**, **f**) the lateral line grooves in transversal view, and (**g**, **h**) the neuromasts in transversal view, for both species, respectively. Cr, cartilaginous rings; E, non-sensory epithelium; Ma, mantle cells; N, nerve bundle; S, sensory (hair) cells; Su, support cells.

In both species, scanning electron microscopy and histology reveal cylindrical grooves, where the lumen is oval or U-shaped in transverse section, with similar diameters and a slightly narrowed opening in both species (Table 2; Figs. 2a and b and 3e and f). Specimens of both species have grooves of a significantly larger diameter on their ventral side (W = 85, *p* < 0.01 for *H. bemisi*; W = 20, *p* = 0.045 for *H. avia*). Anterior grooves are significantly larger in *H. bemisi* than in *H. avia* (*N* = 10, W = 91, *p* = 0.048). *Hydrolagus bemisi* displays anterior grooves of almost 2 mm in diameter, while those in *H. avia* do not exceed 1 mm in diameter. Volumetric and histological data reveal that LLGs are supported by subcutaneous C-shaped cartilaginous rings in both species (Fig. 3). These rings support the grooves, but are absent in the large dilations on the anterior and antero-ventral grooves in *H. bemisi*. Spaces between rings appear larger on the anterior part of the head in this species, although this was not examined quantitatively.

**Table 2:** Diameter of the lateral line grooves (LLG), ampullary pores and ampullary bulbs in both species. Measurements (N, per species) are reported as mean values ± standard deviation (SD).

Species	LLG diameter (µm)	Ampullary pore diameter (µm)	Ampullary bulb diameter (µm)
*Hydrolagus bemisi*	713.89 ± 230.5 (*N* = 20)	564.94 ± 38.50 (*N* = 262)	1434.20 ± 379.55 (*N* = 22)
*Harriotta avia*	515.74 ± 545.49 (*N* = 19)	554.56 ± 63.77 (*N* = 388)	1167.35 ± 289.61 (*N* = 27)

In both species, the few neuromasts that could be unequivocally located and identified using both scanning electron microscopy and histology are oval and elongated along the groove axis, raised into an ovoid, apically-convex shape (when viewed in transverse section), and located in the medial part of the grooves (Figs. 2c-f and 3). From scanning electron micrographs and histological sections, neuromasts are sitting at the bottom of the LLG (Figs. 2a, b, e and f and 3). However, for some ventral samples, both species display neuromasts situated off the midline, at the base of the groove. Neuromasts comprise columnar sensory hair cells, surrounded by support cells (or sustentacular cells), themselves bordered by mantle cells (Figs. 2g and h and 3c-d and g-h). The mantle cells delineate the boundary between neuromasts and the non-sensory epithelium lining the groove walls (Figs. 2g and h and 3c-d and g-h). Each sensory hair cell has a long kinocilium and an adjacent bundle of stereocilia on its apical surface (Fig. 2i, j). The surrounding support cells possess numerous, long microvilli that are not longer than the stereocilia bundles (Fig. 2i, j).

### Gross morphological organisation of the ampullary system

The gross organisation of the ampullary system is similar in both species. Each ampullary pore opening at the skin surface leads to an ampullary bulb via a gel-filled canal. Ampullae are grouped into a single, large, rostral cluster encompassing the whole rostrum (Fig. 1b, c, e, f). The ampullary canals are straight, of various lengths and are orientated in various directions, depending on the distribution of their corresponding pores (Fig. 1b, c, e, f). This main rostral cluster is gelatinous and lacks any visible structures that would show the presence of physical divisions into sub-clusters. The assessment of innervation, i.e., the afferent nerve bundles projecting from different areas of the ampullary cluster to the brain, which could provide more information about any compartementalisation present, was out of the scope of the current study. The rostral cluster in *H. bemisi* encloses most of the rostral ampullae, tapering towards both the mouth ventrally and towards the eyes dorsally. This cluster corresponds to the superficial ophthalmic-buccal cluster of other elasmobranchs. All ampullary canals from cephalic ampullary pores converge into this unique cluster (Fig. 1b, c). Similarly in *H. avia*, the rostral cluster occupies the whole anterior part (two thirds) of its elongated rostrum and is supported by an elongated cartilaginous protrusion (rostral skeletal cartilage) that sits medially and extends longitudinally along the length of the rostrum. The posterior part of the rostral cluster occupies a third of the rostrum’s height between the buccal area and the eyes (Fig. 1e, f).

The distribution of ampullary pores is uneven and reveals different densities across the head (Fig. 1). In both species, pores are concentrated near their cluster around the rostrum and close to the mouth (Fig. 1). This translates to a higher proportion of pores ventrally than dorsally in *H. bemisi* and *H. avia*, with 90% and 80% of their pores located ventrally, respectively (Table 3). Due to the morphological variation of their rostra, *H. bemisi* and *H. avia* have more pores anteriorly, with 87% and 97% of their pores located rostrally, respectively. However, total pore counts are markedly different, as *H. avia* has twice as many pores (1245 ± 137) than *H. bemisi* (600 ± 64, Table 3).

Both species of holocephalans have skin areas naturally defined by the LLGs, which can be used to divide the skin into ‘zones’ where ampullary pore size differ. Both species display up to four distinct pore sizes (Fig. 1). Typically, the largest pores (> 1 mm in diameter), with some exceeding 1 mm in diameter in *H. bemisi* and some reaching almost 2 mm diameter in *H. avia*, are on the posterior-dorsal part of the head. Ventrally, the diameter of pores increases from the nostrils to the tip of the rostrum, with the smallest pores situated in the area rostral to the nostrils and the largest pores situated towards the rostrum tip. Pore diameter ranges from 97.11 µm to 1,678.66 µm in *H. bemisi*, and from 86.46 µm to 2,158.17 µm in *H. avia*. Mean pore diameter is similar within species (564.94 ± 38.50 µm for *H. bemisi*; 554.56 ± 63.77 µm for *H. avia* – Table 2). Dorsal pores in both *H. bemisi* and *H. avia* are significantly larger than the ventral pores (W = 1249, p < 0.0001; W = 2625, p < 0.0001, respectively). There are significant differences in pore size between species on the ventral (W = 33566, p = 0.04), anterior (W = 35303, p = 0.04) and posterior areas (t (59) = 2.4651, p = 0.02). Generally, *H. avia* displays the smallest pores on the centre of its rostrum on the ventral head surface, between the nasal (N) groove and the nostrils (Fig. 1f). The variation in ampullary pore diameter in *H. bemisi* is mostly similar to that of *H. avia*, in that the smallest pores are ventrally located, and pore diameter is greatest on the posterior part of the head.

We observe one or two ‘pore-like’ openings on the dorsal (upper part) and ventral (lower part) sides of the eyeballs in both species (see Supplementary Figure S1). These ‘pore-like’ openings resemble ampullary pores, with comparable external diameters and appearance, but could not be unambiguously assigned to this sensory feature without further investigation. These qualitative observations will require further histological assessments to confirm whether they are sensory in nature.

**Table 3:** Ampullary pore counts for each study species (*n* = 3 specimens), expressed as number ± standard deviation (SD) and percentage of the total count.

Species (*n* = 3)	Total count	Ventral	Dorsal	Anterior	Posterior
*Hydrolagus bemisi*	600 ± 64	542 ± 54 90%	58 ± 11 10%	522 ± 65 87%	78 ± 9 13%
*Harriotta avia*	1245 ± 137	997 ± 111 80%	247 ± 28 20%	1212 ± 146 97%	33 ± 10 3%

### Micro-structure of ampullary bulbs and the sensory epithelium

**Fig. 4:**
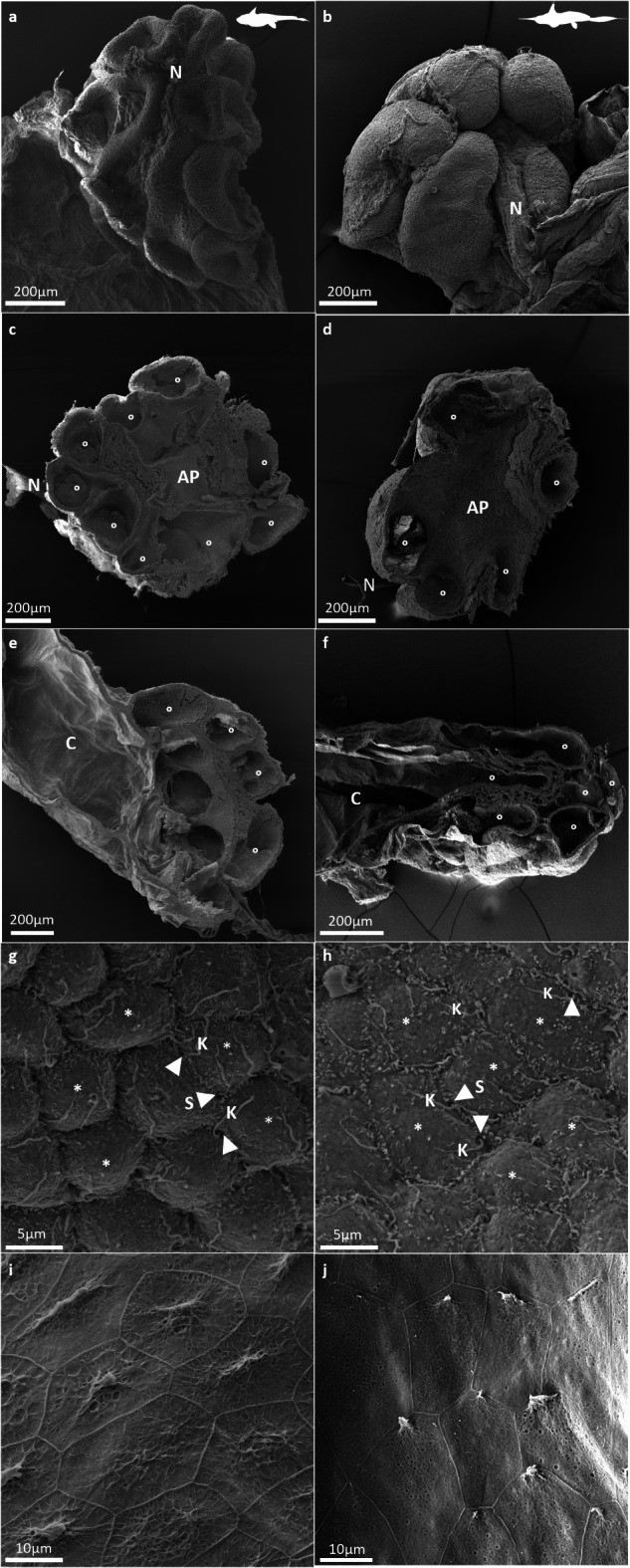
Scanning electron micrographs of the electrosensory organs in each of the two study species – *Hydrolagus bemisi* (left) and *Harriotta avia* (right) – showing: (**a**, **b**) the external surface of an ampullary bulb, with its finger-shaped sensory chambers, part of its associated canal and afferent nerve bundle (N), (**c**, **d**) the internal organisation and structure of an ampullary bulb and several ramified sensory chambers (°) from the ampullae proper (AP), in transversal view, (**e**, **f**) the internal organisation and structure of an ampullary bulb with several ramified sensory chambers (°) from the ampullae proper (AP) with the origin of the associated ampullary canal (C) on the apical side of the ampullary bulb, in longitudinal view, (**g**, **h**) the sensory epithelium composed of electroreceptor cells (S & white arrows), which narrow apical surfaces bear a single long kinocilium (K), surrounded by large, hexagonal support cells (*), which apical surfaces bear a single, short, central primary cilium and several short microvilli; and (**i**, **j**) the ampullary canal walls lined with non-sensory epithelium made up of typical squamous epithelial cells.

**Fig. 5:**
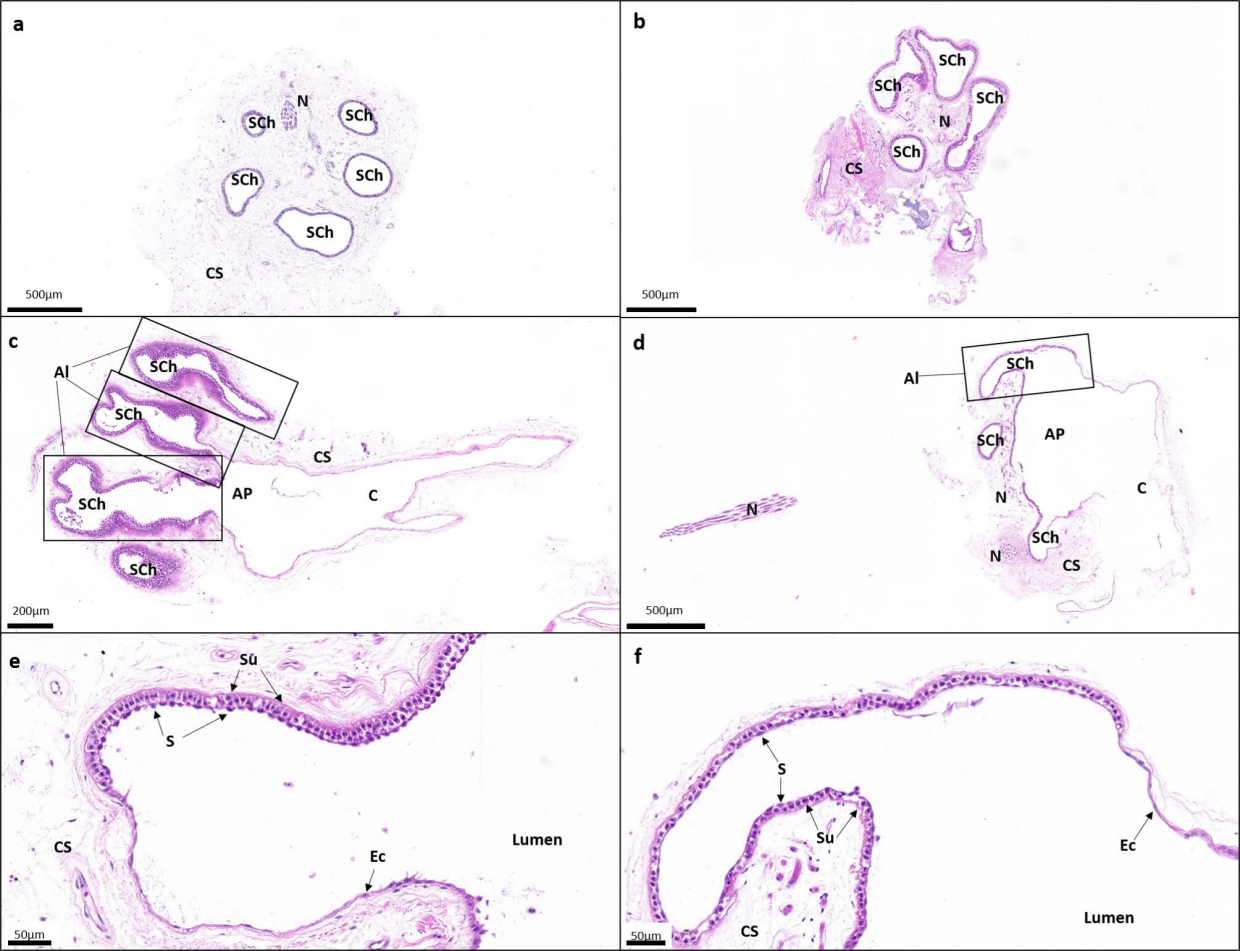
Light micrographs showing a selection of histological sections of the ampullary bulbs and their sensory chambers, stained with a modified solution of Hematoxylin and Eosin-Y, in both study species (left; *Hydrolagus bemisi*; right: *Harriotta avia*). The transversal (**a**, **b**) and longitudinal (**c**-**f**) images show: (**a**, **b**) several sensory chambers (Sch) or alveoli (Al) encapsulated in the collagen sheath (CS) forming the ampullary bulb, (**c**, **d**) the ampullary canal (C) connecting to the ampulla proper (AP) at the entrance of the bulb, which then divides into several, single sensory chambers, and (**e**, **f**) the delineation between non-sensory epithelial walls of the ampullary canal (C) and the ampulla proper (AP), and the sensory epithelium lining the sensory chambers within the ampullary bulb. Ec, non-sensory epithelial cells constituting the canal walls; N, nerve; S, sensory cell; Su, support cell.

Both species have dactyliform (or finger-shaped) ampullae (Fig. 4a, b) grouped into a single, large rostral cluster (Fig. 1b, c, e, f). Ampullary bulbs have 7–9 dactyliform sensory chambers in *H. bemisi* and 4–9 dactyliform sensory chambers in *H. avia*, which are distinguishable using light and electron microscopy (Figs. 4a and b and 5a and b). At the entrance (apex) of the ampullary bulb, the ampullary canal opens into a large ampulla proper leading to several sensory chambers (Figs. 4e and f and 5c and d), each comprising a single alveolus lined with sensory epithelium. At the bottom (base) of the ampullary bulb, nerve fibres emanating from the different alveoli collectively form an afferent nerve bundle (Figs. 4a-d and 5a and b).

There are no subdivisions into alveoli within the chambers; each elongated sensory chamber is characterised by a single alveolus (Figs. 4c-f and 5c and d). Inside the ampulla, the organisation of the epithelial walls is similar between species (Figs. 4g-j and 5e and f). Typically, the canal walls are comprised of a thin non-sensory epithelium of flattened squamous epithelial cells (Figs. 4i and j and 5e and f), while the sensory epithelium lining the sensory chambers is thicker and composed of support cells surrounding the sensory cells (Figs. 4g and h and 5e and f). Within the non-sensory epithelium, cuboidal epithelial cells are of an intermediate cell morphology found between the canal walls and the proximal entrance of the sensory chambers (Fig. 5e, f). Within the sensory epithelial layer, a small part of the sensory cells is directly in contact with the lumen, while support cells that surround them span the entire epithelium thickness (Fig. 5e, f). The apical surface of electrosensory cells is barely visible apart from a single, long kinocilium, which protrudes into the lumen and is visible at the edges of surrounding support cells (Fig. 4g, h). The support cells possess several short microvilli and a single, short, central primary cilium (Fig. 4g, h).

The ampullary bulbs and their canals are surrounded by sheaths of collagenous connective tissue (Fig. 5). The ampullary bulbs are larger in *H. bemisi* than in *H. avia*, with a mean diameter of 1,434.20 ± 379.55 μm compared to 1,167.35 ± 289.61 μm, respectively (W = 423, *p* < 0.02) (Table 2).

## Discussion

The lateral line and electrosensory systems of holocephalans have received little attention but represent two important (non-visual) sensory modalities, which may be especially critical for survival in low light environments, such as the deep sea. As it was hypothesised that there is a correlation between sensory anatomy and ecology, a comparison in the arrangement of these peripheral sense organs could help predict feeding strategies for localising benthic prey and identifying conspecifics, and the relative importance of detecting minute hydrodynamic disturbances and weak electric fields. The following sections focus on establishing structure-function relationships for each of the two sensory modalities based on the anatomical data in order to predict the sensory ecology of the two study species within their aquatic environment.

### Implications for lateral line function

While their structure, distribution and arrangement seem homologous to other chondrichthyans, the presence of lateral line grooves (LLGs) appears to be a unique feature of the lateral line system in deepwater chimaeras, an adaptation that evolved presumably to increase sensitivity to minute hydrodynamic disturbances near the head and along the body. Both study species displayed bilaterally-symmetrical LLGs, which appear to be a sensory adaptation for alerting the presence of food, predator or conspecific in the low light conditions of the deep-sea, given that the lateral line system in shallow water elasmobranchs and callorhinchids (plough-nose chimaeras) possess enclosed, tubular lateral line canals (LLCs)^14,31^. Although grooves (or open lateral line canals) would allow unimpeded stimulation of the neuromasts within them compared to enclosed lateral line canals, their effectiveness as a sensitive system for detecting hydrodynamic disturbances remains to be clarified. Open LLCs occur during canal morphogenesis in bony fishes (at intermediate developmental stages IIb and III), where U- to C-shaped ossified canal walls form on either side of neuromasts^79^, and are retained in the adults of some species (e.g., *Gasterosteus aculeatus*;^80^). Even though LLG number may be conserved among all fishes due to developmental processes, it is likely constrained by the presence of dermal bone in bony fishes. While holocephalans do not have dermal bone, the location and number of LLGs may also be constrained by cartilage and ancestral development processes. The function of lateral and posterior grooves (i.e., trunk, postorbital, supraorbital, infraorbital and supratemporal grooves – Fig. 1a) on the head, which arise from wider and larger grooves on and around the snout in *H. bemisi*, is also not understood. A similar arrangement is found in the closely-related rabbit fish *Chimaera monstrosa*^34^.

The dorso-posterior arrangement of LLGs in both *Hydrolagus bemisi* and *Harriotta avia* have also been described in studies of *C. monstrosa*,* Hydrolagus matallanasi*, and *H. colliei*, where it is suggested that the lateral groove continues along the body, and may be particularly useful for mating and detecting predators^32,81^. The arrangement of LLGs in *H. bemisi* also closely resembles that of *Hydrolagus mccoskeri*, *Chimaera compacta*, and *Hydrolagus matallanasi*, and appears to be common amongst chimaerids (short-nosed chimaeras)^88,83,84^; although the location of the infraorbital, hyomandibular, and angular grooves does vary within this family. Interestingly, like *H. melanophasma*, *H. bemisi* also exhibits some intraspecific variations, which appears to be common among holocephalans^85^.

The distribution of LLGs in *H. avia* is also common to other species of the Rhinochimaeridae, such as *Neoharriotta carri*^86^, *Rhinochimaera africana*^87,88^, *N. pumila*^89^ and *R. pacifica*^90,88^. However, Inada and Garrick (1971)^90^ described a hyomandibular groove originating from the angular groove instead of the infraorbital groove in *H. avia*. The function of lateral line dilations (“circular grooves” or “gland openings” – as per Reese (1910)^32^) in *H. bemisi* is not known. Further investigation is required to determine if these features represent a different type of lateral line organ. *Harriotta avia* exhibited a similar distribution of LLGs to *H. bemisi*, despite its long rostrum. The distribution of ventral LLG in this long-nose chimaera is similar to that in sawsharks and sawfishes^91,92^ (notwithstanding the presence of mandibular, supratemporal, and angular grooves); an observation which would form the basis for a prediction that the two very different groups of chondrichthyans use their sensory systems in similar ways, specifically for the detection of benthic prey. In fact, the rhinochimaerid-like rostrum of sawsharks and sawfishes is thought to assist with the detection of demersal fishes and crustaceans^91–93,94^. Therefore, the potential use of rostral LLGs to detect and locate cryptic, buried prey in rhinochimaerids would be convergent with that of the Pristiophoriformes (sawsharks) and pristid rays (sawfishes).

Neuromasts were located at the basal bottom, medial part of the grooves. As reported for *H. colliei*, some neuromasts were observed in the middle of the larger rostral groove in *H. bemisi*. This observation was also reported for the ventral, thin groove in *H. avia*. Despite the difference of location, the ultrastructure of lateral line neuromast is consistent across both study species. Scanning electron microscopy and light microscopy revealed a linear arrangement of oval neuromasts, similar to those observed in *H. colliei*^32^ and *C. monstrosa*^34^. Transverse sections confirmed the characteristic elongated plateau-shaped structure of neuromasts described by Von Lubitz (1981)^34^. Neuromasts appear similar in structure to those in *C. monstrosa*^34^, with columnar hair cells distributed over the sensory epithelium, and surrounded by large, elongated support cells that span the height of the neuromast, with a ring of mantle cells delineating the neuromast, from its base to its apex^25,34^. The thickness of the non-sensory epithelium within the LLGs, which tapes towards the groove walls, and is comprised of squamous epithelial cells, is similar to previous studies in *C. monstrosa*^34^ and *H. colliei*^32^. Sensory (hair) cells have a single, elongated kinocilium and an adjacent bundle of stereocilia on their apical surface. The sensory cells are surrounded by support cells bearing several microvilli on their apical surface, in both *H. bemisi* and *H. avia*, which aligns with previous descriptions in *C. monstrosa*^34^ and other chondrichthyans^25^. We confirm that neuromast ultrastructure and cell composition are relatively conserved among holocephalan species studied to date, apart from the absence of basal cells (another type of support cells) that were described in *C. monstrosa*^34^, and indicates a common function.

The number and distribution of lateral line canals (LLCs) in elasmobranchs has been suggested to be correlated with habitat and feeding behaviour^95–98^, but could also reflect ancestral evolutionary patterns. Following this hypothesis, the presence of more LLGs ventrally on the rostrum would suggest benthic feeding and confer increased ventral spatial resolution^99^ in both holocephalan species studied. Living in low light conditions at continental slope depths^76,100^, both species feed on benthic organisms^68^ and must rely on a range of non-visual senses to track prey, including detecting small hydrodynamic disturbances using the lateral line system. The presence of enlarged LLGs and dilations on the anterior part of the head in *H. bemisi* may enhance the detection of more cryptic prey^68^; however, no larger nor more numerous neuromasts were observed in these ‘sacculate’ grooves under the scanning electron microscope. These morphological attributes are likely conserved through ancestral patterns.

### Implications for electrosensory function

The structure, arrangement, size and number of electrosensory organs in the two deep-sea chimaeras examined show interspecific differences that reflect the role of electroreception in feeding, predator avoidance and intraspecific social communication. Ampullary pore distribution in both species is comparable to other studies, and appears to correlate with foraging habits^81,83,84^. Piscivorous species, such as the pelagic stingray *Pteroplatytrygon violacea* and the sandbar shark *Carcharhinus plumbeus*, which feed exclusively on pelagic and demersal teleosts tend to have an equal number of pores on the ventral and dorsal sides^43,44,101,102^. While benthic stingrays, such as *Urobatis jamaicensis*, *Neotrygon trigonoides*, and sphyrnid sharks, which feed essentially on benthic prey, have a higher pore number on their ventral side^43,52,58,103^. *Hydrolagus bemisi* and *H. avia* have most of their pores located ventrally (80% and 90%, respectively), similar to *H. colliei*^64^ and *C. monstrosa*^65^, which would be expected for benthivorous holocephalans^71,84^. The high proportion of pores on the ventral side of the elongated rostrum in *H. avia*, coupled with its presumed limited ventral visual field based on head morphology and the lateral placement of the eyes, predicts the need to detect the weak electric fields of benthic organisms in this species. The low number of dorsal pores likely assists in detecting the uniform electric fields of approaching predators^45,56,59,62,101,104,103^.

The number of ampullary pores is also known to be associated with foraging strategy and feeding behaviour in chondrichthyans^44,92,101^. Mean pore number for *H. avia* (1,245 ± 137) is relatively high for an holocephalan. Similar pore numbers occur in the southern sawshark *Pristiophorus nudipinnis* (1,283)^91,94^ and the dwarf sawfish *Pristis clavata* (1,267)^105^. This reinforces the hypothesis that *H. avia* uses its rostrum to locate infaunal prey^91,93,94,105^. The high pore number in *H. avia* could also be an example of a species that fulfils the ‘enhanced electrosensory hypothesis’ postulated for some species of shovelnose rays (Rhinobatidae)^57^ and hammerhead sharks (Sphyrnidae)^103^. The elongated rostrum of rhinochimaerids, compared to the chimaerids, likely requires a higher pore number to maintain pore density, and thus spatial resolution, to successfully locate prey^101^. Jordan et al.. (2009b)^44^ also demonstrates that benthic feeders with a high density of electrosensory pores have a greater resolution to locate the weak electric fields of prey. Mean pore number in *Hydrolagus bemisi* (600 ± 64) lies within the known range for chondrichthyans, from 148 pores in the hornshark *Heterodontus francisci*^106^ to 3,943 pores in the daggernose shark *Carcharinus oxyrhynchus*^107^, and corresponds to that found in other chimaeras, such as *H. colliei* (~ 500)^64^ and *C. monstrosa* (~ 702)^65^. *H. bemisi* has a similar diet, and a comparable number and distribution of ampullary pores as *C. monstrosa*^65^, which is an opportunistic suction feeder^2,65,68^. Although low numbers of ampullary pores have also been associated with either ram or suction-feeding in other (planktivorous) chondrichthyan species^53,108^, we suspect that *H. bemisi* could be a suction feeder, like *C. monstrosa*, mainly based on its diet. Raschi (1986)^101^ proposed that the number of pores in skates would decrease with depth and habitat complexity, whereby deepwater dwellers should have fewer ampullary pores. Interestingly, based on studies by Fields et al. (1993)^63,36^, Bottaro (2022)^65^ and our study, the opposite appears to occur in chimaeras, with deeper dwelling species possessing a high number of ampullary pores than their shallow water counterparts.

Both species of chimaeras possess pores of four different sizes, with the largest pores (> 1 mm in diameter) on the posterior-dorsal part of the head. Pore size decreases anteriorly until the mid-rostrum, which is common in other chimaeras, with the smallest pores found ventrally just above the mouth^63,65^. The functional implications of the inverse relationship between pore size and pore number on the ventral surface of the head in chimaeras remains unclear. However, a high electrosensory resolution for prey localisation is predicted, as has previously been proposed for benthic stingrays^58,59^.

Clusters of ampullary bulbs are described based solely on their location, since their innervation was not examined. Cluster distribution in both species of chimaeras does not follow the typical pattern found in elasmobranchs^47,53,54,55,57,58,59,62,91,109,107,108^, as all the canals emanating from the ampullary pores over the different regions of the head converged onto a single cluster. This single, large cluster fills the rostrum from the anterior buccal area on the ventral side to the anterior part of the eye, and could be described as a superficial ophthalmic-buccal cluster. The cluster of *H. bemisi* is smaller due to the smaller size of its rostrum.

Both study species possess dactyliform (or finger-shaped) ampullae, similar to those described for *C. monstrosa*^65^ and *H. colliei*^63,64^. This characteristic seems to be unique to holocephalans^14,63,65^. However, holocephalan ampullae are homologous to those of elasmobranchs, with the main canal terminating at the apical part of the ampulla sac, the afferent nerve being located at the basal part between the sensory chambers, and the ampulla surrounded by an electrically-insulating collagen sheath^14,26^. Multi-alveolate ampullae occur in both species, a common feature in elasmobranchs, which have up to seven sensory chambers^50,110,61,53,56,97,98^. However, holocephalans have higher numbers of sensory chambers: *C. monstrosa* (8–9;^65^), *H. bemisi* (7–9; this study), and *H. avia* (4–9, but mostly 4–6; this study). There appears to be an inverse relationship between ampulla size and density, which would ultimately affect spatial electrosensory resolution, i.e. *H. bemisi* has fewer pores but larger ampullae (and presumably a high number of electroreceptive neurons), while *H. avia* has substantially more pores but smaller ampullae.

Both species of chimaera have a thin, non-sensory epithelium comprised of a double layer of squamous epithelial cells forming the canal wall, and a thick, sensory epithelium comprised of sensory hair cells surrounded by support cells, an arrangement which is common to all chondrichthyans^25,54,58,65,112–114^. At the transition between the canal wall and the sensory epithelium lie cuboidal epithelial cells, which also occur in elasmobranchs^54^. These epithelial cells are tightly joined to reduce signal loss between the pore and the ampulla proper beneath the skin^25,112,115^. The support cells of the sensory epithelium produce the glycoprotein gel, which is vital for the rapid conduction of weak electric signals and electroreceptor function^23^. In both study species, electroreceptor cells have a single, apical kinocilium, which is a common feature among chondrichthyans^14,63,54,114,115^. An assessment of the anatomy and ultrastructure of the sensory epithelium requires further attention since previous studies have been limited to only a few species of chondrichthyan^59,112,115^, including the eastern shovelnose ray *Aptychotrema rostrata*^113^, the bull shark *Carcharhinus leucas*^114^, and the spotted ratfish *Hydrolagus colliei*^64^.

## Conclusions

Overall, the similar anatomical and topographic arrangement of the peripheral regions of the lateral line and electrosensory systems of *Hydrolagus bemisi* and *Harriotta avia* is likely to reflect the importance of detecting and localising benthic prey that are subsequently ingested by suction feeding. The elongated rostrum of *H. avia* is likely used as a sensory probe, like the saw of Pristiophoriformes^91,94^ and pristid rays^92,109,105^, providing spatially-resolved information about minute hydrodynamic disturbances and the weak electric fields emanating from potential prey situated on or within the substrate. The arrangement of lateral line organs and electroreceptors in *H. bemisi* differs, especially in the number of ampullary pores, suggesting that this species may rely less on electroreception than the lateral line to detect prey, predators and conspecifics. More quantitative analyses of the afferent input (number of axons) and the relative size of the sensory brain regions receiving lateral line and electroreceptive information in each species may provide further insights about the relative importance of these two sensory modalities. Ultimately, such knowledge could be useful in assessing their vulnerability to future anthropogenic disturbances (i.e., laying of oceanic cables and future deep-sea mining activities) in their natural habitats.

## Electronic supplementary material

Below is the link to the electronic supplementary material.

Supplementary Material 1

## Data Availability

All data generated and analysed for this research are either presented in this manuscript or available in Supplementary information. The raw data used for analyses remain available upon request to the corresponding author, Dr. Victoria Camilieri-Asch (victoria.camilieriasch@qut.edu.au).
