# Supplementary material for: The lateral line and electrosensory systems of two holocephalans
Source: Sci Rep. 2025 Feb 28;15:7163. doi: 10.1038/s41598-025-87499-2 (PMC11871310; doi:10.1038/s41598-025-87499-2)
Supplement: Supplementary file 1 — Supplementary Material 1 [file 41598_2025_87499_MOESM1_ESM.docx]

**SUPPLEMENTARY MATERIAL**

**Table S1.** Protocol used for Haematoxylin and Eosin-Alcoholic or Eosin-Y (H and E) staining.

| Step | Station | Solution | Time (min) |
| --- | --- | --- | --- |
| 1 | Oven | 60C | 08:00 |
| 2 | 1 | Xylene | 03:00 |
| 3 | 2 | Xylene | 03:00 |
| 4 | 3 | 100% Ethanol | 02:00 |
| 5 | 4 | 90% Ethanol | 01:00 |
| 6 | 5 | 70% Ethanol | 01:00 |
| 7 | Wash 2 | Water | 01:00 |
| 8 | 8 | Harris Haematoxylin | 04:00 |
| 9 | Wash 5 | Water | 01:00 |
| 10 | 6 | 0.5% Acid Alcohol | 00:01 |
| 11 | Wash 4 | Wash and Blue | 05:00 |
| 12 | 13 | 70% Ethanol | 01:00 |
| 13 | 12 | 0.25% alcoholic eosin | 00:03 |
| 14 | 14 | 90% Ethanol | 00:30 |
| 15 | 15 | 100% Ethanol | 00:30 |
| 16 | 16 | 100% Ethanol | 00:30 |
| 17 | 17 | Xylene | 02:30 |
| 18 | 18 | Xylene | 02:30 |
| 19 | Exit | Xylene |  |

**Table S2.** Terminology of lateral line grooves used among holocephalans and elasmobranchs to date. The names of lateral line grooves vary, sometimes drastically, between studies. In this table, we associate the nomenclature used in our study with that of other studies published to date, to facilitate comparisons. See full references provided below.

| **Name in our study** | **Acronym** | **Name of the groove in other studies** |
| --- | --- | --- |
| Angular ^1-15^ | An | - Holocephalans: oral^4,7-13,15^ or horizontal^6^   (for the posterior part)   - Elasmobranchs: mandibular^19^, hyomandibular^22^ |
| Hyomandibular^5^ | H | - Holocephalans: jugular^1-4^, preopercular^6-15^ - Elasmobranchs: hyomandibular^16,17,19-22^ |
| Infraorbital^9-11,14^ | I | - Holocephalans: orbital^1-4^, postorbital^5^, otic^6,8,12-15^ - Elasmobranchs: infraorbital^16,17,19-22^, postorbital^18^ |
| Mandibular^5,7,8,15^ | M | - Holocephalans: oral^1-4,6^ - Elasmobranchs: mandibular^17-20^ |
| Nasal^1,5-15^ | N | - Holocephalans: angular loop^2,3^, nasal loop^4^ - Elasmobranchs: nasal^18-20^, ventral supraorbital^22^ |
| Postorbital^6,7,9-12,14^ | PO | - Holocephalans: occipital^1-5,8,12-15^ - Elasmobranchs: infraorbital^16-18,22^, postorbital^19^, posterior^20^ |
| Prenasal^18,19,22^ | PN | - Holocephalans: subrostral^1,4,6,8,13^, posterior branch of the suborbital^2^, angular^3^, supraorbital^5,7^, rostral^9-12,4^ (for one part of it) - Elasmobranchs: prenasal^18,19,22^, median^20^ |
| Rostral^1^ | R | - Holocephalans: suborbital^8^, supraorbital^13^   (does not apply for studies 2-7 & 15)   - Elasmobranchs: supraorbital^16,19,20,21^ |
| Suborbital^1-4^ | Sb | - Holocephalans: Infraorbital^5-9,12-15^ - Elasmobranchs: Infraorbital^16-19,21^, dorsal supraorbital^22^ |
| Supratemporal^5-15^ | ST | - Holocephalans: aural^1-4^ - Elasmobranchs: supratemporal^17-20^, posterior lateral line^22^ |
| Supraorbital^5-7,13,15^ | SO | - Holocephalans: cranial^1-4^, suborbital^8^ - Elasmobranchs: supraorbital^16-21^, dorsal supraorbital^22^ |
| Trunk^6-8^ | T | - Holocephalans: lateral^1-5^, main trunk^9-14^ - Elasmobranchs: posterior^16,18-22^, trunk^17^ |

Holocephalans

1. Reese, A. M. The lateral line system of *Chimaera colliei*. *Journal of Experimental Zoology,***9**(2), 349-370 (1910).
2. Bullis Jr, H. R., & Carpenter, J. S. *Neoharriotta carri*: a new species of Rhinochimaeridae from the Southern Caribbean Sea. *Copeia*, 443-450. (1966).
3. Garrick, J. A. F. Harriotta raleighana, a long-nosed chimaera (Family Rhinochimaeridae), in New Zealand waters. *Journal of the Royal Society of New Zealand*, **1**(3-4), 203-213 (1971).
4. Inada, T., & Garrick, J. A. F. Rhinochimaera pacifica, a long-snouted chimaera (Rhinochimaeridae), in New Zealand waters. *Japanese Journal of Ichthyology,***25**(4), 235-243 (1979).
5. Compagno, L. J. V., Stehmann, M., & Ebert, D. A. *Rhinochimaera africana*, a new longnose chimaera from southern Africa, with comments on the systematics and distribution of the genus Rhinochimaera Garman, 1901 (Chondrichthyes, Chimaeriformes, Rhinochimaeridae). *South African Journal of Marine Science,***9**(1), 201-222 (1990).
6. Didier, D. A., & Stehmann, M. *Neoharriotta pumila*, a new species of longnose chimaera from the northwestern Indian Ocean (Pisces, Holocephali, Rhinochimaeridae). *Copeia*, 955-965 (1996).
7. Didier, D. A., & Nakaya, K. Redescription of Rhinochimaera pacifica (Mitsukuri) and first record of R. africana Compagno, Stehmann & Ebert from Japan (Chimaeriformes: Rhinochimaeridae). *Ichthyological Research,***46**, 139-152 (1999).
8. Soto, J. M., & Vooren, C. M. Hydrolagus matallanasi sp. nov.(Holocephali, Chimaeridae) a new species of rabbitfish from southern Brazil. *Zootaxa*, **687**(1), 1-10 (2004).
9. Barnett, L. A., Didier, D. A., Long, D. J., & Ebert, D. A. *Hydrolagus mccoskeri* sp. nov., a new species of chimaeroid fish from the Galapagos Islands (Holocephali: Chimaeriformes: Chimaeridae).*Zootaxa,***1328**(1), 27-38 (2006).
10. Quaranta, K. L., Didier, D. A., Long, D. J., & Ebert, D. A. (2006). A new species of chimaeroid, Hydrolagus alphus sp. nov.(Chimaeriformes: Chimaeridae) from the Galapagos Islands. *Zootaxa*, **1377**(1), 33-45 (2006).
11. James, K. C., Ebert, D. A., Long, D. J., & Didier, D. A. A new species of chimaera, Hydrolagus melanophasma sp. nov.(Chondrichthyes: Chimaeriformes: Chimaeridae), from the eastern North Pacific.*Zootaxa*, **2218**(1), 59-68 (2009).
12. Kemper, J. M., Ebert, D. A., Naylor, G. J., & Didier, D. A. Chimaera carophila (Chondrichthyes: Chimaeriformes: Chimaeridae), a new species of chimaera from New Zealand. *Bulletin of Marine Science*, **91**(1), 63-81 (2014).
13. Clerkin, P. J., Ebert, D. A., & Kemper, J. M. New species of Chimaera (Chondrichthyes: Holocephali: Chimaeriformes: Chimaeridae) from the Southwestern Indian Ocean. *Zootaxa*,**4312**(1), 1-37 (2017).
14. Iglésias, S. P., Kemper, J. M., & Naylor, G. J. Chimaera compacta, a new species from southern Indian Ocean, and an estimate of phylogenetic relationships within the genus Chimaera (Chondrichthyes: Chimaeridae). *Ichthyological Research*, 1-15 (2022).
15. Finucci, B., Didier, D., Ebert, D. A., Green, M. E., & Kemper, J. M. H arriotta avia sp. nov. a new rhinochimaerid (Chimaeriformes: Rhinochimaeridae) described from the Southwest Pacific. *Environmental Biology of Fishes*, 1-25 (2024).

Elasmobranchs

1. Maruska, K. P. Morphology of the mechanosensory lateral line system in elasmobranch fishes: ecological and behavioral considerations. *Environmental Biology of Fishes*, **60**(1), 47-75. (2001).
2. Jørgensen, J. M., & Pickles, J. O. The lateral line canal sensory organs of the epaulette shark (Hemiscyllium ocellatum). *Acta Zoologica*, **83**(4), 337-343 (2002).
3. Marzullo, T. A., Wueringer, B. E., Jnr, L. S., & Collin, S. P. Description of the mechanoreceptive lateral line and electroreceptive ampullary systems in the freshwater whipray, Himantura dalyensis. *Marine and freshwater research*, **62**(6), 771-779 (2011).
4. Theiss, S. M., Collin, S. P., & Hart, N. S. The mechanosensory lateral line system in two species of wobbegong shark (Orectolobidae).*Zoomorphology*, **131**, 339-348 (2012).
5. Winther-Janson, M., Wueringer, B. E., & Seymour, J. E. Electroreceptive and mechanoreceptive anatomical specialisations in the epaulette shark (Hemiscyllium ocellatum). *PLoS One*,**7**(11), e49857 (2012).
6. Shibuya, A., Zuanon, J., & de Carvalho, M. R. Neuromast distribution and its relevance to feeding in Neotropical freshwater stingrays (Elasmobranchii: Potamotrygonidae). *Zoomorphology*, **139**(1), 61-69 (2020).
7. Wueringer, B. E., Winther‐Janson, M., Raoult, V., & Guttridge, T. L. Anatomy of the mechanosensory lateral line canal system and electrosensory ampullae of L orenzini in two species of sawshark (fam. P ristiophoridae). *Journal of Fish Biology*, **98**(1), 168-177 (2021).

**
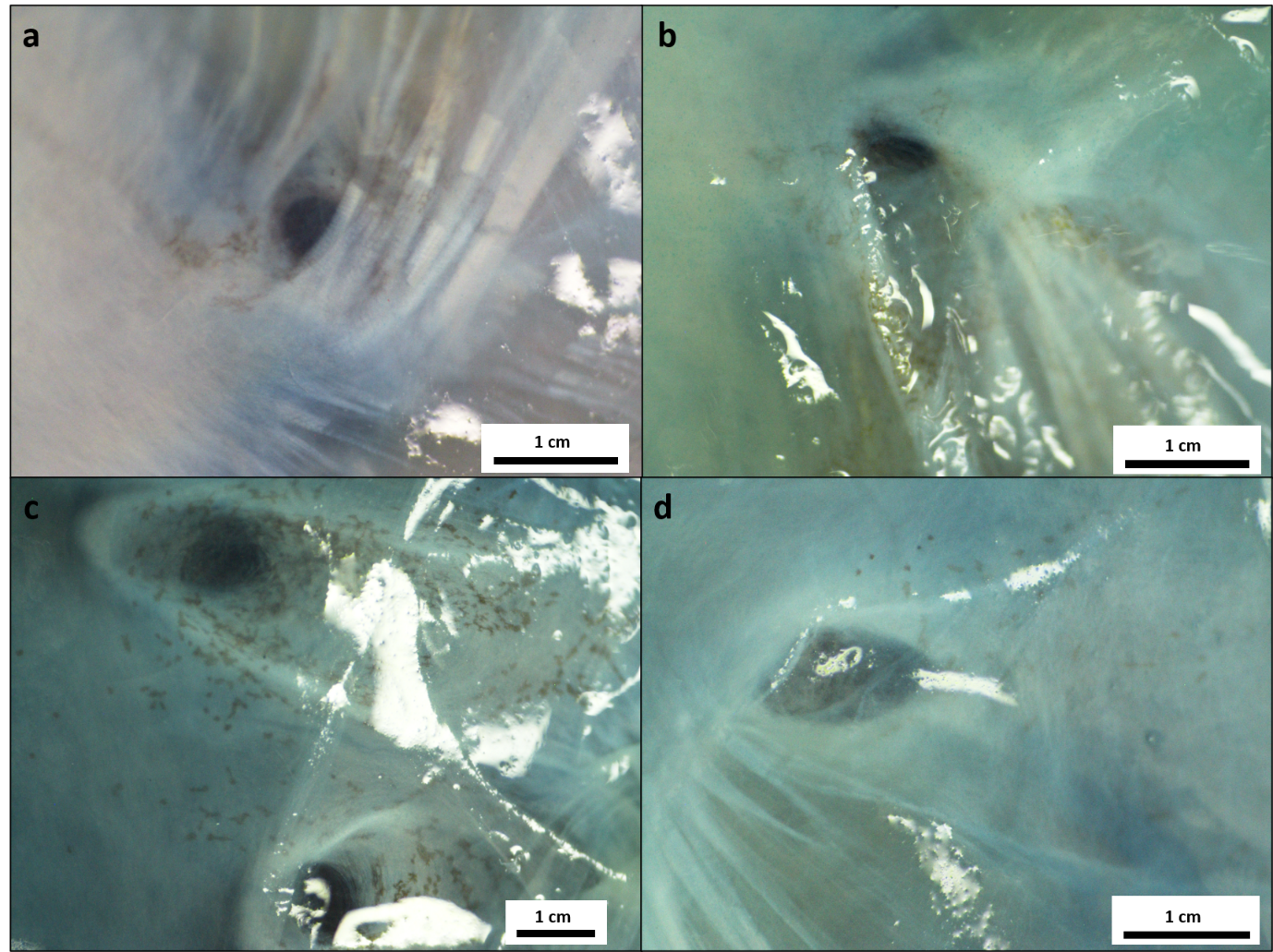
**

**FIGURE S1**. Light micrographs showing the presence of ‘pore-like’ openings observed on the left (a, c) and right (b, d) eyeballs of *Hydrolagus bemisi* (a, b) and *Hariotta avia* (c, d). These openings are present on both the dorsal (upper part) and ventral (lower part) of the eyeballs in both species.
